# Mexiletine as a treatment for primary erythromelalgia: normalization of biophysical properties of mutant L858F Na_V_1.7 sodium channels

**DOI:** 10.1111/bph.12788

**Published:** 2014-08-29

**Authors:** R Cregg, J J Cox, D L H Bennett, J N Wood, R Werdehausen

**Affiliations:** 1 Molecular Nociception Group, Wolfson Institute for Biomedical Research, UCLLondon, UK; 2 UCL Centre for Anaesthesia, Critical Care and Pain MedicineLondon, UK; 3 The Nuffield Department of Clinical Neuroscience, University of OxfordUK; 4 Department of Anesthesiology, Medical faculty, Heinrich-Heine-UniversityDüsseldorf, Germany

## Abstract

**Background and Purpose:**

The non-selective sodium channel inhibitor mexiletine has been found to be effective in several animal models of chronic pain and has become popular in the clinical setting as an orally available alternative to lidocaine. It remains unclear why patients with monogenic pain disorders secondary to gain-of-function SCN9a mutations benefit from a low systemic concentration of mexiletine, which does not usually induce adverse neurological side effects. The aim of this study was, therefore, to investigate the biophysical effects of mexiletine on the L858F primary erythromelalgia Na_V_1.7 mutation *in vitro*.

**Experimental Approach:**

Human wild-type and L858F-mutated Na_V_1.7 channels were expressed in HEK293A cells. Whole-cell currents were recorded by voltage-clamp techniques to characterize the effect of mexiletine on channel gating properties.

**Key Results:**

While the concentration-dependent tonic block of peak currents by mexiletine was similar in wild-type and L858F channels, phasic block was more pronounced in cells transfected with the L858F mutation. Moreover, mexiletine substantially shifted the pathologically-hyperpolarized voltage-dependence of steady-state activation in L858F-mutated channels towards wild-type values and the voltage-dependence of steady-state fast inactivation was shifted to more hyperpolarized potentials, leading to an overall reduction in window currents.

**Conclusion and Implications:**

Mexiletine has a normalizing effect on the pathological gating properties of the L858F gain-of-function mutation in Na_V_1.7, which, in part, might explain the beneficial effects of systemic treatment with mexiletine in patients with gain-of-function sodium channel disorders.

## Table of Links

**Table:** 

TARGETS	LIGANDS
NaV	Mexiletine
NaV1.7	Lidocaine

This Table lists key protein targets and ligands in this document, which are hyperlinked to corresponding entries in http://www.guidetopharmacology.org, the common portal for data from the IUPHAR/BPS Guide to PHARMACOLOGY (Pawson *et al*., 2014) and are permanently archived in the Concise Guide to PHARMACOLOGY 2013/14 (Alexander *et al*., 2013).

## Introduction

The non-selective sodium channel inhibitor mexiletine hydrochloride [1-methyl-2-(2,6-xylyloxy)ethylamine hydrochloride] has been extensively studied and used clinically for decades because of its antiarrhythmic effects (Chew *et al*., 1979; Woosley *et al*., 1984; Fenster and Comess, 1986; Monk and Brogden, 1990). More recently, it has also been found to be effective in several animal models of chronic pain and it has been suggested as a third-line treatment instead of systemic lidocaine for neuropathic pain syndromes (Jarvis and Coukell, 1998; Kuhnert *et al*., 1999; Mao and Chen, 2000; Challapalli *et al*., 2005; Tremont-Lukats *et al*., 2005; Ebell, 2006; Marmura, 2010). Based on its almost complete absorption after oral administration, its low first-pass effect and a plasma elimination half-life of approximately 10–14 h, it is also an orally available alternative to lidocaine for systemic treatment (Middleton, 1980).

**Figure 1:**
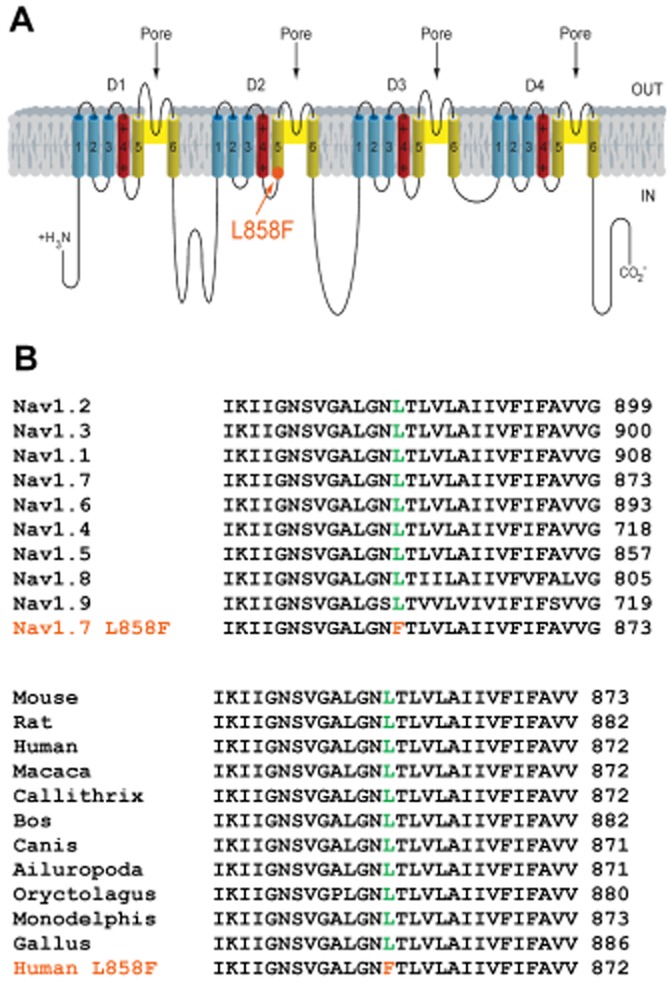
(A) Diagram of Na_V_1.7 showing the location of the p.L858F amino acid substitution. p.L858F maps to the S4–S5 intracellular linker of domain 2 and is identified by the orange solid circle and the arrow. (B) Amino acid residue alignment around p.L858F. Amino acid residue alignment demonstrating the magnitude of inter-isoform and inter-species conservation of the affected regions. Orange residues identify the mutant p.L858F mapping to the S4–S5 linker of DII of the human Na_V_1.7α subunit. Green colour indicates the corresponding non-mutated variant.

Recently, it has been noted that systemic treatment with mexiletine alleviates the symptoms and signs of myotonia in non-dystrophic myotonia (Statland *et al*., 2012). Non-dystrophic myotonia results from mutations in the voltage-gated sodium channel encoded by the gene *SCN4A* (Matthews *et al*., 2010). In comparison, gain-of-function mutations in the related *SCN9A* gene, which encodes Na_V_1.7, can result in primary erythromelalgia (PEM), either a familial or a sporadic chronic neuropathic pain syndrome (Dib-Hajj *et al*., 2005). Several publications have reported an analgesic effect of mexiletine in patients suffering from this monogenic pain disorder (Kuhnert *et al*., 1999; Legroux-Crespel *et al*., 2003; Dib-Hajj *et al*., 2005; Nathan *et al*., 2005; Choi *et al*., 2009). The apparent absence of pro-arrhythmic and other major adverse effects at analgesic doses of mexiletine points to a mode of action that preferentially affects pathological channels. While a pronounced use-dependent sodium channel block in an erythromelalgia-causing Na_V_1.7 mutation has been demonstrated before (Choi *et al*., 2009), the mechanisms that lead to the observed analgesic effect are not fully understood. The L858F mutation in Na_V_1.7 is one of the best characterized and most common gain-of-function mutations that leads to PEM and is associated with a severe phenotype (Han *et al*., 2007; Samuels *et al*., 2008; Cheng *et al*., 2011; Segerdahl *et al*., 2012). The 858 residue is located near the pore region within the second domain of Na_V_1.7 (Figure 1A). Sequence alignment of voltage-gated sodium channels (Na_V_s) shows that the leucine at position 858 is individually conserved in every member of the Na_V_ family in humans and highly evolutionarily conserved in all Na_V_1.7 homologues (Figure 1B). It has previously been shown that the L858F mutation causes a hyperpolarizing shift in the voltage- dependence of steady-state fast activation and an enhanced response to slow depolarizations (Han *et al*., 2006).

Therefore, the aim of this study was to investigate the biophysical effects of mexiletine on the PEM-causing L858F Na_V_1.7 mutation *in vitro*, with particular focus on channel gating properties. A better understanding of how mexiletine affects the gating properties of a pathologically dysfunctional channel may enable research to focus on specific gating properties of this channel for future drug development. Thus, we expressed human Na_V_1.7 channels with or without the L858F mutation in HEK293A cells and analysed the effect of mexiletine on their whole-cell voltage-clamp properties.

## Methods

### Plasmid and site-directed mutagenesis

A previously described full-length human SCN9A cDNA sequence cloned into a modified pcDNA3 expression vector containing downstream polio IRES and DsRED2 sequences (FLRED) was used (Cox *et al*., 2006). The L858F mutation was introduced into FLRED using the QuikChange II XL Site-Directed Mutagenesis Kit (Stratagene, La Jolla, CA, USA) according to the manufacturer’s instructions. The coding sequence of both constructs was fully sequenced to verify the desired mutation and to ensure that no other variations had been introduced.

### Cell culture and transfection

HEK293A cells were cultured in a humidified atmosphere containing 5% carbon dioxide at 37°C and were grown in DMEM (Gibco, Life Technologies, Carlsbad, CA, USA) supplemented with 10% heat-inactivated FBS. Unless stated otherwise, reagents were purchased from Sigma Aldrich (St. Louis, MO, USA).

Cells were transiently transfected with plasmid DNA for expression of Na_V_1.7 human wild-type (WT) α subunits (Ref Seq NM_002977) or L858F-mutated α subunits (SCN9A-IRES-DsRed2 in pcDNA3 vector) combined with human WT β1 (Ref Seq NM_001037) and β2 (Ref Seq NM_001037) subunits (SCN1B-IRES-SCN2B-IRES-eGFP in a pIRES2-AcGFP1 backbone vector) as previously described (Cox *et al*., 2006). In brief, transient transfection was performed with cells seeded at 80–90% confluency in 35 mm cell culture dishes using Lipofectamine 2000 (Invitrogen, Life Technologies) according to the manufacturer’s recommendations. After 6 h, the transfection medium was replaced with fresh culture medium and the cells re-seeded for electrophysiological recordings at 20–30% confluency.

### Electrophysiological recordings

Whole-cell membrane current recordings were performed 46–78 h after transfection. All recordings were made at room temperature. Micropipettes were pulled from borosilicate glass capillaries (GC150F-10; Harvard Apparatus, Kent, UK) using a Brown-Flaming P-97 horizontal micropipette puller (Sutter Instruments, Novato, CA, USA, USA) and then fire polished on a microforge (MF-830 Narishige Group, Tokyo, Japan). Voltage errors were minimized with correction and prediction mode of series resistance compensation, both set to 50%. Extracellular (bath) solution contained (in mmol·L^−1^): 140 NaCl, 4 KCl, 2 CaCl_2_, 1 MgCl_2_, 10 HEPES, adjusted to pH 7.4 with NaOH, osmolarity 320–325 mOsm·L^−1^ with glucose. Pipettes were filled with an intracellular solution containing (in mmol·L^−1^): 140 CsCl, 5 NaCl, 5 EGTA, 2 MgCl_2_, 10 HEPES adjusted to pH 7.3 with CsOH, osmolarity 305–310 mOsm·L^−1^ with glucose. Once filled with the appropriate intracellular solution, recording electrodes had a resistance between 2.0 and 3.2 MΩ. A silver chloride-coated silver wire served as a reference electrode with one end connected to the ground input of the amplifier and the tip placed directly into the bath solution. Cells having a leak current, after establishment of a whole-cell configuration, of more than 10% of the peak sodium current were discarded and those which had developed a leak current of this magnitude during the experiment were not used in the final analysis. The liquid junction potentials between the bath and the pipette solutions were not corrected. Whole-cell membrane currents were filtered at 5 kHz and sampled at 20 kHz using an Axopatch 200B patch clamp amplifier (Molecular Devices, Foster City, CA, USA) and Digidata 1200B A/D (analogue to digital) converter (Molecular Devices). Data were acquired on a Windows-based PC using Clampex (Molecular Devices) software and analysed by pCLAMP (Clampfit) 9.2 software (Molecular Devices).

### Mexiletine treatment

Mexiletine hydrochloride salt was dissolved in extracellular (bath) solution to give a stock solution of 100 mM and pH was adjusted to 7.4. Subsequent dilutions were performed in a standard external solution to reach the desired concentrations. Addition of mexiletine did not change the osmolarity of the extracellular solution used in these experiments. For microperfusion with laminar flow at 1 mL·min^−1^, a gravity-driven perfusion system (MEV-9, BioLogic Science Instruments, Claix, France) equipped with polyethylene tubing was used. The solution was removed using a Dymax 5 suctioning apparatus (Charles Austen Pumps Ltd., Byfleet, Surrey, UK). For concentration–response experiments, mexiletine was applied in a stepwise approach from the lowest to highest concentration, and 30 s was allowed after each concentration change for the response to reach a plateau before the effect induced was recorded.

### Voltage-clamp protocols

To characterize the voltage-dependence of the steady-state channel activation, currents were evoked by voltage increments of 10 mV from −80 to +40 mV for 10 ms from a holding potential of −120 mV with 5 s between pulses. Conductance (*G*) values were calculated from the peak inward currents (*I*) measured and the reversal potential for sodium ions (*E*_rev_ Na) observed using the equation *G* = *I*/(*V*_m_ − *E*_rev_ Na).

Reversal potential was measured by extrapolating the linear portion of the *I*/*V* relationship between +10 and +40 mV. The resulting values for conductance were normalized to the peak conductance and fitted using the Boltzmann equation. Steady-state inactivation of WT Na_V_1.7 channels or L858F mutant channels was assessed by holding cells at increasing potentials from −110 to 0 mV for 500 ms, followed by a step to −10 mV for 50 ms.

For characterization of the use-dependence of mexiletine-induced block in Na_V_1.7 WT and L858F variant channels, currents were elicited in whole-cell configuration by initially holding the cell at −120 mV and stepping up to 0 mV for 5 ms every 200 ms, generating pulses at a frequency of 5 Hz.

### Statistical analysis

All data are expressed as means ± SEM. Differences in means between WT channels and mutations were tested by Student’s two-tailed *t*-test or one-way anova with the Bonferroni post test where appropriate. *P* < 0.05 was considered significant. Calculations were made using the GraphPad Prism software version 5.0 (GraphPad Software Inc., La Jolla, CA, USA).

## Results

**Figure 2:**
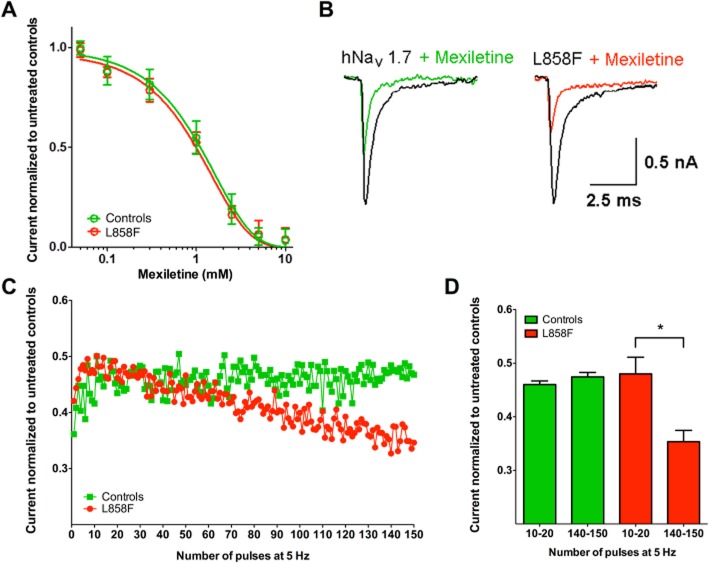
Concentration-dependent and use-dependent effects of mexiletine on Na_V_1.7-mediated currents in HEK293 in whole-cell voltage-clamp configuration. (A) Concentration-dependent reduction in peak current by mexiletine in wild-type Na_V_1.7 control channels and L858F channel mutation. Non-linear regression, calculated by plotting log-transformed inhibitor versus normalized response, generated IC_50_ of 1.1 mM (0.05) for wild-type Na_V_1.7 channels and 0.87 mM (0.06) for L858F mutant channels. (B) Representative examples of current traces before and during treatment with mexiletine (500 M) at the last depolarizing voltage step of a high-frequency stimulation protocol with 150 pulses at 5 Hz as described below. (C/D) Peak currents were normalized to maximum currents in untreated controls. Currents were elicited in whole-cell configuration by initially holding the cell at −120 mV and stepping up to 0 mV for 5 ms every 200 ms, generating pulses at a frequency of 5 Hz. While mexiletine (500 μM) led to a use-dependent fall-off in peak current for L858F, no use-dependent effect was found in controls with wild-type Na_V_1.7 channels. All data are presented as means ± SEM (*n* = 8). One-way anova and *post hoc* Bonferroni test; *P < 0.05.

HEK293A cells transfected with the Na_V_1.7 α subunit containing the L858F mutation (*n* = 35) in combination with the Na_V_β1 and Na_V_β2 subunits did not differ significantly from cells with WT Na_V_ 1.7 (*n* = 29) in terms of peak current densities and whole-cell capacity and series resistance (Table 1). The inhibition of WT and L858F channels by mexiletine was assessed by measuring the magnitude of the reduction in peak current achieved by depolarizing cells to 0 mV from a resting potential of −120 mV. Both peak currents (WT and L858F) were reduced in the presence of mexiletine in a concentration-dependent manner (Figure 2A). The fit of concentration–response curves with a first-order binding function revealed that the half-maximal blocking concentration (IC_50_) was 1.1 ± 0.05 mM for the WT and 0.87 ± 0.06 mM for the L858F mutant channels.

**Table 1:** Cell and recording properties

	Peak current (nA)	Whole-cell capacity (pF)	Series resistance (MΩ)
Controls (*n* = 29)	−0.68 (0.09)	16.8 (1.88)	12.7 (1.22)
L858F (*n* = 35)	−0.53 (0.08)	22.7 (1.90)	16.4 (1.99)
*P* value	0.08 (ns)	0.12 (ns)	0.11 (ns)

Data presented as mean values and SEM. Differences in means of cells with Na_V_1.7 L858F mutant channels compared with Na_V_1.7 wild-type controls were tested by Student’s two-tailed *t*-test. *P* < 0.05 was considered significant.

Channels with the L858F mutations demonstrated a greater amount of use-dependent normalized peak current fall-off when compared with the WT in the presence of mexiletine (500 μM) when depolarized at a frequency of 5 Hz (Figure 2B and C). Comparing averaged currents recorded between pulse 10 and 20 with currents recorded between pulse 140 and 150, normalized peak current for cells expressing channels with the L858F mutation were reduced by 26% (*n* = 8; *P* < 0.05), while the effect of mexiletine on currents recorded from WT controls remained unchanged (Figure 2D).

**Figure 3:**
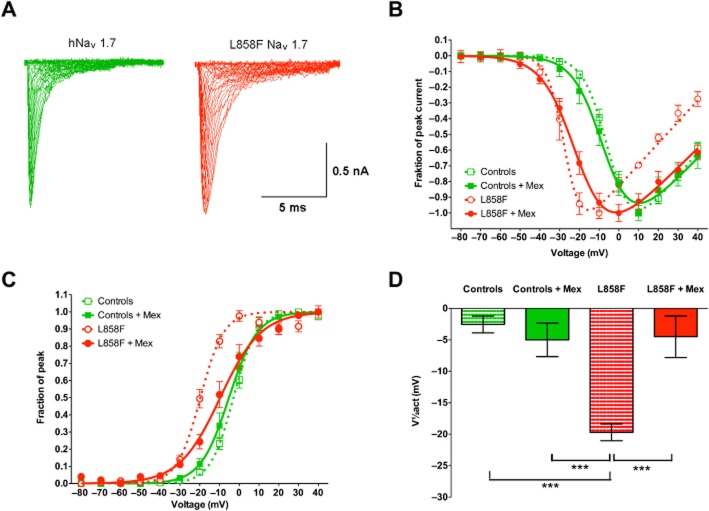
Voltage-dependence of steady-state activation of Na_V_1.7-mediated currents in whole-cell voltage-clamp configuration. (A) Representative current traces recorded from HEK293A cells that expressed wild-type (left) or L858F mutant (right) Na_V_1.7 together with hβ1 and hβ2. Whole-cell Na^+^ currents were elicited by 10 ms test pulses with 5 s intervals to potentials between −80 and 40 mV in steps of 10 mV from a holding potential of −120 mV. (B) Current–voltage plot of wild-type Na_V_1.7 (Controls), wild-type Na_V_1.7 channels exposed to 500 μM mexiletine (Controls + Mex), L858F mutation (L858F) and L858F mutation exposed to 500 μM mexiletine (L858F + Mex). (C) Conductance values from HEK293 cells expressing wild-type Nav1.7 (Controls), wild-type Na_V_1.7 channels exposed to 500 μM mexiletine (Controls + Mex), L858F mutants (L858F) and L858F mutants exposed to 500 μM mexiletine (were calculated from peak inward currents in activation protocols, normalized and fitted using the Boltzmann equation). (D) Analysis of voltages for half-maximal activation (V_1/2__act_) revealed a hyperpolarizing shift in the voltage-dependence of steady-state activation in L858F-expressing cells compared with controls. Mexiletine treatment of L858F-expressing cells, however, shifted the current-voltage relationship towards wild-type values, although a complete return to physiological values was not achieved. Please note that the mean data as used for statistical analysis do not entirely correspond to the graph depicted in panel (B) as these were fitted from averaged experimental results to demonstrate one representative graph for display purposes. All data presented are as means ± SEM; *n* = 20 for controls; *n* = 9 for controls exposed to mexiletine, *n* = 15 for L858F mutation and *n* = 20 for L858F mutation exposed to mexiletine. One-way anova with *post hoc* Bonferroni test; ***P < 0.001.

In Na_V_1.7 channels with the L858F mutation, the voltage-dependence of channel activation was found to be changed to more hyperpolarizing potentials compared with WT channels when normalized peak inward currents were plotted as a function of depolarization potential to demonstrate the current–voltage relationship (Figure 3A and B). When the resulting conductance values were fitted to a Boltzmann equation (Figure 3C), the L858F mutation caused a hyperpolarizing shift in the voltage-dependence of steady-state activation (Figure 3D; *P* < 0.01), with a voltage step leading to half-maximal activation (V_1/2act_) of −19.7 ± 1.3 mV (*n* = 20) compared with −2.6 ± 1.3 mV (*n* = 15) in WT controls (*P* < 0.01). While mexiletine (500 μM) did not have an effect on the voltage-dependence of steady-state activation in WT Na_V_1.7 channels (−5.0 ± 2.7 mV; *n* = 9), it shifted those of channels with the L858F mutation towards physiological values (−4.5 ± 3.3 mV) (Figure 3D; *n* = 20; *P* < 0.01). Furthermore, in L858F mutant channels, the slope of the function describing the voltage–conductance relationship was significantly changed by mexiletine (4.6 ± 0.7 vs. 8.5 ± 1.2; *P* < 0.001; Figure 3C), whereas it remained unchanged in Na_V_1.7 WT channels (5.1 ± 0.2 vs. 5.4 ± 0.4).

**Figure 4:**
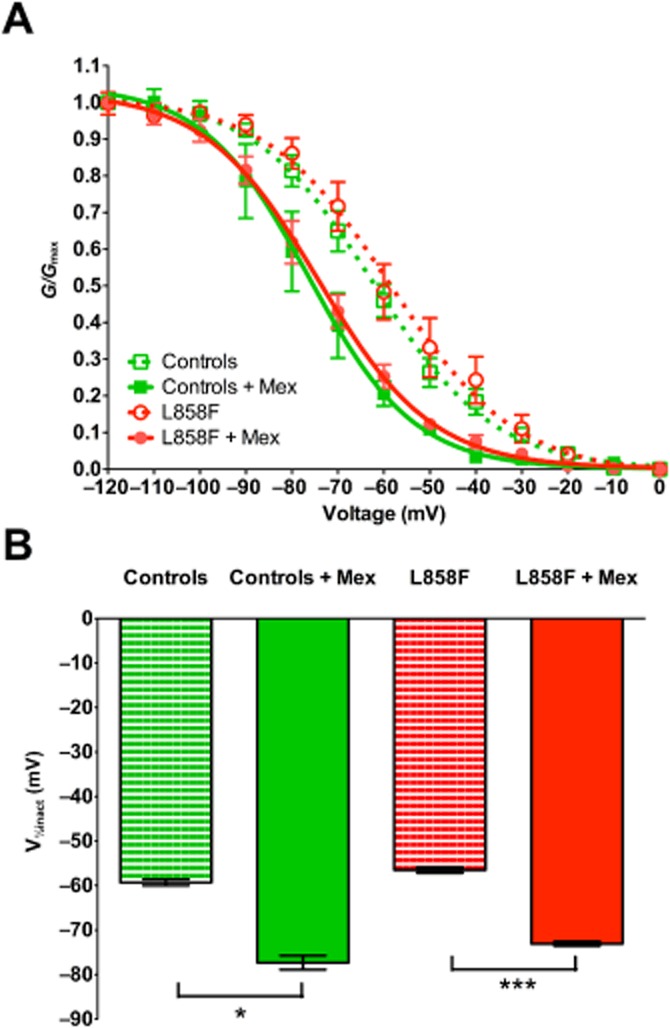
Voltage-dependence of steady-state fast inactivation of Na_V_1.7-mediated currents in whole-cell voltage-clamp configuration. (A) Voltage-dependent inactivation in HEK293 cells expressing wild-typeNa_V_1.7 (Controls), wild-type Na_V_1.7 channels exposed to 500 μM mexiletine (Controls + Mex), L858F mutants (L858F) and L858F mutants exposed to 500 μM mexiletine (L858F + Mex) was assessed by holding the cells at potentials increasing from −120 to 0 mV for 500 ms, followed by a step to −10 mV for 50 ms, normalized to peak currents and fitted using the Boltzmann equation. (B) Analysis of voltages for half-maximal inactivation (V_1/2inact_) in L858F-expressing cells and wild-type Na_V_1.7 controls. A similar degree of hyperpolarizing shift in inactivation curves was observed in L858F mutant- and the wild-type Na_V_1.7-expressing cells exposed to 500 μM mexiletine. Slope factors of the fitted curves did not differ between L858F mutants and wild-type Na_V_1.7. All data are presented as means ± SEM; *n* = 7 for controls; *n* = 5 for controls exposed to mexiletine, *n* = 19 for L858F mutation and *n* = 12 for L858F mutation exposed to mexiletine. One-way anova with *post hoc* Bonferroni test; *P < 0.05; ***P < 0.001.

When analysing the voltage-dependence of the steady-state fast inactivation (Figure 4), we found no difference in the voltages with half-maximal inactivation (V_1/2inact_) between WT channels and those with the L858F mutation (−59.3 ± 3.1 and −56.5 ± 2.5 mV respectively). Mexiletine (500 μM) shifted the V_1/2inact_ towards more hyperpolarized potentials for Na_V_1.7 WT and L858F mutant channels (−77.3 ± 4.7 mV; *n* = 14; *P* < 0.01 and −73.0 ± 2.2 mV; *n* = 15; *P* < 0.01 respectively). The slope factors of fitted curves did not differ between L858F mutants and WT Na_V_1.7.

**Figure 5:**
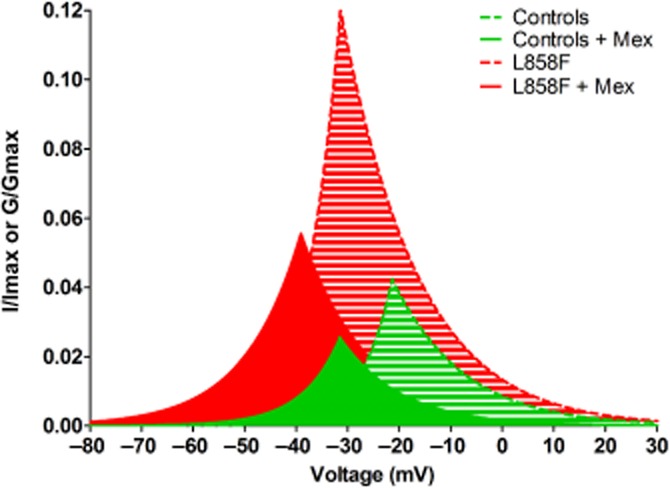
Analysis of window currents in whole-cell voltage-clamp configuration of Na_V_1.7-expressing HEK293 cells. Combined superimposed fitted curves of steady-state activation and fast inactivation kinetics in cells expressing Na_V_1.7 channels (controls or L858F mutant) with Na_V_β1 and Na_V_β2 subunits. Window currents of cell population expressing wild-type channels (green striped area) were reduced by mexiletine (500 μM; green solid area). Mexiletine treatment (500 μM; red solid area) also reduced window currents of cells expressing L858F mutant channels (red striped area). All data are presented are as means.

A composite graph showing Boltzmann fits of mean activation and steady-state inactivation curves of L858F mutant versus WT channels showed an increase in the window current (Figure 5). Maximum window currents for WT Na_V_1.7 channels were 4.5% of the peak currents (AUC = 0.82) as opposed to 11.5% seen in the mutant channel population of cells (AUC = 2.49). Following the application of mexiletine (500 μM) and after a steady state had been reached, there was a reduction in the maximum window current to 5.5% of peak currents in L858F channels and a reduction in the window current AUC by 48% (AUC = 1.29).

## Discussion

PEM is a devastating human disorder in which patients suffer from excruciating episodic pain, mainly in the hands and feet. A frequently used treatment for this disorder is the sodium channel blocker mexiletine. Here, we have studied the concentration- and frequency-dependent effects of mexiletine on Na_V_1.7 channels with the erythromelalgia-associated mutation L858F, whose effect on channel properties as has been described previously (Han *et al*., 2007; Cheng *et al*., 2011). Intriguingly, mexiletine caused a change in the gating properties of this mutated channel, leading to an almost completely normalized voltage-dependence of activation and window currents, whereas the voltage-dependence of the steady-state fast inactivation was shifted to more hyperpolarized potentials in both the WT and mutant L858F channels to a similar degree. This effect substantially contributed to the reduction in the windows currents observed after treatment with mexiletine in L858F mutant channels. Although the effects of mexiletine have been investigated previously in another (V872G) Na_V_1.7 mutation *in vitro*, where it was shown to induce use-dependent current blocking effects (Choi *et al*., 2009), to our knowledge, this is the first evidence that the mechanism of action of mexiletine on erythromelalgia channel mutations involves an effect on their gating properties.

Furthermore, we observed that mexiletine induced a use-dependent inhibition of peak currents in the L858F mutant channels. A likely explanation for this phenomenon has been suggested by Waxman and co-workers (Choi *et al*., 2009) when investigating another mutant channel. V872G was tested in similar conditions with high-frequency stimulation pulsing to −10 mV in the presence and absence of mexiletine. A stronger, use-dependent fall-off of current can be explained by a hyperpolarizing shift in activation of the mutant channel. Thus, a greater proportion of mutant channels will be opened and then inactivated during the early part of action potential generation. Once reaching +20 mV, activation of both V872G as well as WT channels is at its maximum. At −10 mV, however, it is possible for us to observe this effect. When comparing the use-dependent effect to tonic block, we also think that a frequency-dependent interaction between L858F and mexiletine may be the main reason why this drug, as well as other sodium channel blockers, is effective in PEM.

In a recent study of a gain-of-function mutation (M1476I) in Na_V_1.4 (Zhao *et al*., 2012), which is associated with cold-induced myotonia, effects on channel gating properties were also observed, although mexiletine significantly shifted the voltage-dependent activation and inactivation curves of both WT and M1476I channels in a hyperpolarizing direction. In contrast, the voltage-dependence of activation in Na_V_1.7 L858F channels was shifted in a depolarizing direction, whereas wild-type channels remained unaffected. In agreement with their findings, we found a hyperpolarizing shift in steady-state fast inactivation. Subtype-specific effects on voltage-gating properties of Na_V_ have been found before, for example, for lidocaine (Chevrier *et al*., 2004), which only shifts steady-state inactivation, but not activation in Na_V_1.7, while both activation and inactivation were affected in Na_V_1.8.

It should be noted that the concentration of mexiletine found to be effective in the experiments described is much higher than expected, based on the therapeutic concentrations active clinically. This may, in part, be explained by the simplified nature of the experiments, with cells highly overexpressing the investigated channel, acute wash-in of mexiletine rather than the slow accumulation that would in neuronal tissues *in vivo*, and the experiments were performed at lower ambient temperatures. A further reason may be related to the use-dependence of the drug, since in our experiments the membrane was held at a potential of −120 mV to remove steady-state inactivation. This may have resulted in a reduction in the blocking potency due to the unavailability of channels that would normally be open at the resting membrane potential of nociceptive neurons. Indeed, a reduction in the blocking potency of the same drug has already been described for voltage-gated sodium channels in skeletal muscle (Courtney, 1981). Nevertheless, the higher IC_50_ values for the channel blocking actions of sodium channel blockers observed is very common for this type of experimental approach, as recently demonstrated for lidocaine (Sheets *et al*., 2011).

Other medications that have been demonstrated to be of benefit in erythromelalgia patients have been previously investigated *in vitro* and responsiveness to treatment has been shown to be dependent on the exact causative mutation*.* For example, carbamazepine has been shown to normalize the hyperpolarizing shift in the voltage-dependence of activation produced by the V400M or S241T Na_V_1.7 mutations but have no effect on the F1449V mutation (Fischer *et al*., 2009; Yang *et al*., 2012). Similar effects on the voltage-dependence of steady-state fast inactivation have been observed previously for carbamazepine, phenytoin and lamotrigine in WT voltage-gated sodium channels in rat cultured cortical neurons (Errington *et al*., 2008).

A further example of a substance with effects on voltage-gating properties of wild-type Na_V_1.7 is the sea anemone peptide blood-depressing substance I (BDS-I), which was found to shift the voltage-dependence of both activation and inactivation towards more depolarized potentials. Furthermore, the authors also reported a change in the slope factor of the activation curve, an effect that we also observed for mexiletine in the L858F mutant channels (Liu *et al*., 2012).

More recently, Wu and colleagues characterized three patients clinically diagnosed and confirmed to host EM-causative mutations in their *SCN9A* genes. Causative mutations were overexpressed in CHO cells, and IC_50_ values of lidocaine and mexiletine were compared with the mutations identified as I136V, I848T and V1316A (Wu *et al*., 2013). They found differential effects between lidocaine and mexiletine, which were similar to the treatment effects observed in the affected patients.

Despite the almost complete normalization of activation properties in our case and tendency to normalization of repetitive firing current characteristics, a complete diminishment of the phenotypic aberrations affecting the sufferers of this rare but extremely debilitating painful disorder has not yet been reported. As recently proposed (Estacion *et al*., 2011; Waxman, 2013), PEM symptoms might result from interactions between sympathetic and sensory neurons and so treatment modalities focused on altering neuronal excitability in the dorsal root ganglia alone may be either ineffective or only partially beneficial. Although mexiletine can provide some pain relief for erythromelalgia patients, it is by no means a cure for all. By understanding how mexiletine alters the biophysical properties of specific mutant Na_V_1.7 channels, such as the one investigated in this study, we can potentially find new and better ways to normalize these biophysical properties and hopefully ameliorate pain.

## Conclusions

In summary, our study demonstrates an example of predicting the treatment effect of mexiletine in patients suffering from a specific gain-of-function mutation in Na_V_1.7. By applying the same *in vitro* approach to other PEM mutations, it might be possible to provide an informed prediction of the usefulness of mexiletine as a treatment for this rare inherited pain disorder.

## Abbreviations

A/D: analogue to digital
*E*_rev_: reversal potential
*I*: current
Na_V_: voltage-gated sodium channel
PEM: primary erythromelalgia
WT: wild type
